# Glucagon-like peptide-1 receptor agonist use prior to spinal surgery results in reduced postoperative length of stay: A propensity-score matched analysis

**DOI:** 10.1016/j.xnsj.2025.100612

**Published:** 2025-04-18

**Authors:** Samuel N. Goldman, Kyle Mani, Thomas Scharfenberger, Emily Kleinbart, Aaron T. Hui, Rafael De la Garza Ramos, Mitchell S. Fourman, Ananth S. Eleswarapu

**Affiliations:** a Department of Orthopaedic Surgery, Albert Einstein College of Medicine, Bronx, NY, United States; b Department of Neurological Surgery, Montefiore Einstein, Bronx, NY, United States; c Department of Orthopaedic Surgery, Montefiore Einstein, Bronx, NY, United States

**Keywords:** Spinal fusion, Semaglutide, Length of stay, GLP-1 agonists, Spine surgery, Diabetes mellitus, Obesity

## Abstract

**Background:**

While glucagon-like peptide-1 receptor agonists (GLP-1 RAs) have demonstrated benefits in reducing complications following total knee and hip arthroplasty, their effects in spinal surgery remain unclear. Prior studies have reported mixed results across select spinal fusion procedures, and the impact of GLP-1 RAs on perioperative outcomes has not been well-defined. This study evaluates the association between preoperative GLP-1 RA use and key perioperative outcomes in spinal surgery.

**Methods:**

We conducted a retrospective, propensity score-matched analysis of adult patients (≥18 years) undergoing spinal decompression and/or fusion at an urban academic spine service over the past 5 years. Patients prescribed a GLP-1 RA preoperatively comprised the exposure cohort. A 1:4 nearest-neighbor propensity score matching algorithm was then used to identify comparable controls without GLP-1 RA use, based on age, sex, body mass index (BMI), primary procedure code, comorbidities (diabetes, hypertension, hyperlipidemia, heart disease, smoking status, kidney disease, and anxiety), and the use of prevalent diabetic medications (insulin, metformin, sulfonylureas, and SGLT-2 inhibitors). Primary outcomes included length of stay (LOS), operating room (OPR) time, 90-day reoperation, 90-day readmission, and nonroutine discharge. Binary outcomes were assessed using multivariate logistic regression, while the Mann-Whitney U test was used for continuous variables.

**Results:**

The final matched cohort included 1385 patients (GLP-1 RA: *n* = 277; control: *n* = 1,108), with anterior cervical discectomy and fusion being the most common procedure (*n* = 333, 24%). The GLP-1 RA cohort was predominantly female (*n* = 172, 62.1%), with a mean age of 61.4 years and mean BMI of 33.8 kg/m². Both cohorts exhibited high rates of comorbidities, including diabetes and hypertension. GLP-1 RA use was associated with a significant reduction in median postoperative LOS (3 days vs. 4 days; p = 0.036), particularly among patients undergoing lumbar fusion. No significant differences were observed in OPR time, 90-day reoperation, 90-day readmission, or nonroutine discharge rates.

**Conclusions:**

Preoperative GLP-1 RA use was associated with a statistically significant reduction in postoperative LOS among patients undergoing spinal decompression and/or fusion. Further prospective, multi-institutional studies are warranted to validate these findings and to determine whether this reduction translates into clinically and financially meaningful benefits, including improved long-term outcomes.

## Introduction

The rising global prevalence of diabetes mellitus (DM) and obesity necessitates understanding the impact of medications used in the management of these comorbid conditions on orthopedic outcomes [1]. Approximately 80% of patients undergoing spinal surgery are overweight, obese, or have DM [2], conditions associated with higher rates of postoperative infections, venous thromboembolism, impaired wound healing, and revision surgeries [3], leading to increased costs [4], and worse clinical outcomes [5,6].

Optimizing preoperative management of DM and obesity improves spinal surgery outcomes [7]. For example, morbidly obese individuals who undergo bariatric surgery prior to thoracolumbar spinal procedures experience lower rates of deep vein thrombosis (DVT), neurologic complications, and implant-related complications compared to those without bariatric surgery [8]. Similarly, tight glycemic control in diabetic patients, achieved through interventions like lowering HbA1c% below 6% [[9], [10], [11]] or using metformin [12], is associated with improved patient outcomes and reduced costs following spinal surgery. However, certain DM medications, such as thiazolidinediones (TZDs) and sodium-glucose cotransporter-2 inhibitors (SGLT-2i), have been implicated in impaired bone formation and altered calcium-phosphate homeostasis, potentially worsening spinal fusion outcomes [13].

Glucagon-like peptide-1 receptor agonists (GLP-1 RAs), increasingly utilized for the management of DM and obesity, effectively lower blood sugar and reduce hunger [14,15] by stimulating insulin secretion, suppressing glucagon secretion, and delaying gastric emptying [7,16]. GLP-1 RAs promote substantial weight loss, enhance glycemic control, and reduce the risk of myocardial infarction, stroke, and cardiovascular-related death in diverse patient groups, including those with type 1 and 2 DM and obese/overweight individuals [[17], [18], [19], [20]].

Notably, preoperative semaglutide use has been associated with reduced complications, readmissions, and prosthetic joint infections in total knee and hip arthroplasty (TKA/THA) [21,22]. Recent studies have investigated GLP-1 RA use in spine surgery, yielding mixed results. Seddio et al. [23] reported reduced 90-day adverse events following single-level posterior lumbar fusion, while Tao et al. [24] found no significant differences in short-term outcomes following cervical decompression and fusion. More broadly, Wiener et al. [25] observed lower infection rates, fewer revisions, and improved quality of life among a large cohort of spinal fusion patients using GLP-1 RAs. However, the effects of GLP-1 RAs on perioperative and in-hospital outcomes—such as length of stay (LOS), operative (OPR) time, and discharge disposition—remain poorly characterized.

This study aims to address this gap by analyzing the association between preoperative GLP-1 RA use and perioperative outcomes in patients undergoing spinal decompression and/or fusion. Using a 1:4 propensity-score matched analysis, we compared LOS, OPR time, 90-day reoperation, 90-day readmission, and nonroutine discharge rates between patients prescribed GLP-1 RAs and matched controls. We hypothesized that GLP-1 RA use would be associated with superior perioperative outcomes.

## Methods

### Patient selection and cost variables

Ethical approval for this retrospective study was obtained from the Albert Einstein College of Medicine Institutional Review Board. Data was acquired from urban academic Orthopaedic surgery and Neurological surgery spine services electronic health records (EHRs). Adult patients (≥18 years) who underwent spinal decompression and/or fusion between January 10th, 2018, and September 28th, 2023 were included. Patients undergoing surgery for trauma or infectious indications were excluded to focus on elective cases. The exposure group consisted of patients prescribed a GLP-1 receptor agonist (GLP-1 RA) prior to surgery, including any FDA-approved formulation during the study period, namely dulaglutide, semaglutide, liraglutide, and exenatide. A list of the 15 most common procedures performed within the cohort is presented in Table 1.

**Table 1:** Patient counts, stratified by top 15 most prevalent procedures for the overall cohort.

Procedure	n (%)
Anterior cervical discectomy and fusion/Instrumentation	333 (24.0)
Laminectomy lumbar fusion/Instrumentation open	224 (16.2)
Laminectomy lumbar open	161 (11.6)
Transforaminal lumbar interbody fusion/Instrumentation open	118 (8.5)
Posterior cervical fusion/Instrumentation without laminectomy open	86 (6.2)
Posterior lumbar fusion/Instrumentation open without laminectomy	51 (3.7)
Laminectomy lumbar minimally invasive	46 (3.3)
Laminectomy cervical fusion/Instrumentation	45 (3.2)
Posterior lumbar interbody fusion/Instrumentation	40 (2.9)
Laminectomy thoracic	35 (2.5)
Anterior cervical corpectomy fusion/Instrumentation	34 (2.5)
Transforaminal lumbar interbody fusion/Instrumentation minimally invasive	29 (2.1)
Posterior thoracic fusion/Instrumentation open without laminectomy	21 (1.5)
Posterior thoracolumbar fusion with or without osteotomy	20 (1.4)
Oblique lumbar interbody fusion	19 (1.4)

### Propensity score matching algorithm

A 1:4 propensity score matching algorithm was used to minimize confounding and improve comparability between the GLP-1 RA and control cohorts. Matching was conducted on the following covariates: age, sex, body mass index (BMI), primary procedure code, and comorbidities (diabetes, hypertension, hyperlipidemia, heart disease, smoking status, kidney disease, and anxiety), as well as the use of insulin, metformin, sulfonylureas, and SGLT-2 inhibitors. Propensity scores were calculated using a logistic regression model, and the nearest neighbor method without replacement was applied to identify matched pairs, utilizing the MatchIt package in R (R Software, Vienna, Austria).

To assess the effectiveness of the matching process, standardized mean differences (SMD) were calculated for each covariate both before and after matching, as SMD is the most widely used, and easily comprehensible, metric for evaluating matching quality [26]. An SMD of less than 0.1 was considered ideal, while values less than 0.2 were deemed acceptable and indicative of negligible differences between groups, reflecting adequate covariate balance. The bal.tab function from the cobalt package in R was used for this assessment, and the results were visualized through a Love plot to graphically represent the covariate balance before adjustment and the covariate balance achieved by the matching procedure (Figure).

**Figure:**
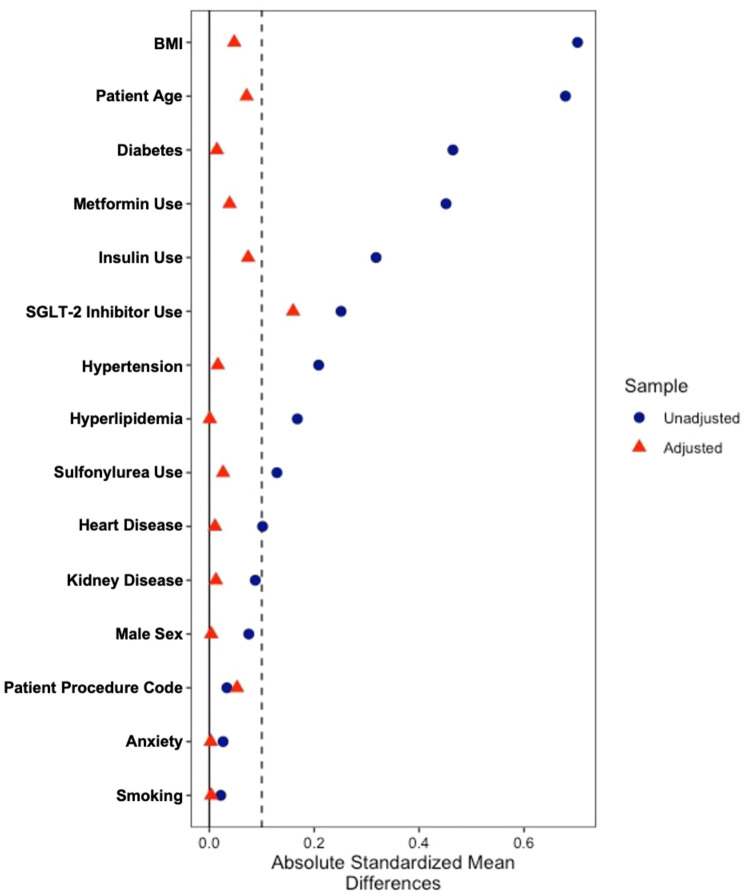
This love plot illustrates the standardized mean differences (SMD) of covariates before (blue circles and after (red triangles) propensity score matching. A threshold of 0.1 SMD (represented by the red dashed lines) is used to denote ideal balance. Covariates with SMD values close to 0 and less than 0.10 are considered well-balanced. The covariates include procedure type, sex, age, BMI, diabetes, hypertension, hyperlipidemia, heart disease, smoking, kidney disease, anxiety, insulin use, metformin, SGLT2 Inhibitor, and sulfonylureas. Matching improves balance when the red triangles approach the zero line.

### Sensitivity analyses

Sensitivity analyses were conducted to evaluate the robustness of the results to potential unmeasured confounding. This included examining the impact of varying the matching ratio, exploring ratios up to 1:5, which was the maximum possible given the available patient data. Ultimately, the 1:4 ratio was chosen for the primary analysis due to its balance between achieving adequate covariate balance and maximizing the number of matched pairs.

### Study outcomes and data analyses

Multivariate logistic regression was used to analyze binary outcomes, including 90-day reoperation, 90-day readmission, and nonroutine discharge rate with odds ratios (ORs), 95% confidence intervals (CIs), and associated p-values reported (significance threshold: p < .05).

For continuous outcomes, including LOS and OPR time, the Shapiro-Wilk test confirmed non-normal distribution (p < .05), prompting the use of nonparametric analyses. Between-group comparisons were conducted using the Mann-Whitney U test, with medians and interquartile ranges (IQRs) reported as the primary summary statistics. To provide additional clinical context, means, standard deviations, and linear regression results were also calculated, allowing estimation of the approximate percentage reduction in LOS associated with GLP-1 RA use.

### Subcohort analysis

Given the inherent heterogeneity of spinal procedures, we performed both a primary analysis on the overall matched cohort and a stratified analysis across procedural subgroups. These subgroups combined similar procedures and included: (1) anterior cervical discectomy and fusion (ACDF) combined with anterior cervical corpectomy and fusion (ACCF); (2) posterior cervical fusion (PCF); (3) lumbar laminectomy (open and minimally invasive); and (4) lumbar fusion, including open and minimally invasive transforaminal lumbar interbody fusion (TLIF) posterior lumbar interbody fusion (PLIF), oblique lateral interbody fusion (OLIF), extreme lateral lumbar interbody fusion (XLIF), and anterior lumbar interbody fusion (ALIF). Outcome measures were assessed within both the overall cohort and these procedural subgroups.

## Results

### Demographic characteristics

A total of 1385 patients were included in the analysis (277 who used GLP-1 RAs, and 1108 matched controls). Of the 277 individuals using GLP-1 RAs, 122 used injectable semaglutide (44%), 108 (39%) used injectable dulaglutide, 36 (13%) used injectable liraglutide, 7 used oral semaglutide (Rybelsus, 2.5%), and 4 used exenatide (1.4%). The top 3 most prevalent procedures were (1) ACDF (*n* = 333, 24%), (2) laminectomy lumbar fusion/instrumentation open (*n* = 224, 16.2%), and (3) laminectomy lumbar open (*n* = 161, 11.6%, Table 1).

Table 2 presents baseline patient counts, stratified by clinical covariates and demographics, along with the respective SMDs for each matched variable, indicating minimal variation between the groups. The distribution of sex was similar between the cohorts, with females representing 62.1% of the GLP-1 RA group and 62.5% of the matched controls (SMD = 0.4%). The mean age of patients was comparable between the groups, with the GLP-1 RA group having a mean age of 61.4 years (SD = 9.1) and matched controls having a mean age of 62.0 years (SD = 12.1). The mean BMI was 33.8 kg/m² in the GLP-1 RA cohort and 33.5 kg/m^2^ in the control group (SMD = 4.7%).

**Table 2:** Clinical covariates and demographical information stratified by GLP-1 RA utilization.

Clinical covariates	GLP-1 agonist n (%)	No GLP-1 agonist n (%)	Standardized mean difference (%)*
Demographics	*n* = 277	*n* = 1108	
Mean age (years)	61.4	62.0	−7.1
Sex			0.4
Male	105 (37.9)	416 (37.5)	
Female	172 (62.1)	692 (62.5)	
Mean BMI (Kg/m²)	33.8	33.5	4.7
Comorbidities			
Diabetes	198 (71.5)	808 (72.9)	−1.4
Hypertension	162 (58.5)	666 (60.1)	−1.6
Hyperlipidemia	98 (35.4)	391 (35.3)	0
Cardiovascular disease	53 (19.1)	200 (18.1)	1.1
Kidney disease	46 (16.6)	170 (15.3)	1.3
Anxiety	34 (12.3)	133 (12.0)	0.3
Medication use			
Insulin use	112 (40.4)	366 (33.0)	7.4
SGLT-2 inhibitor use	77 (27.8)	131 (11.8)	16.0
Sulfonylurea use	47 (17.0)	159 (14.4)	2.6
Metformin use	166 (59.9)	621 (56.0)	3.9
Prevalent procedures			5.3
ACDF	60 (21.7)	273 (24.6)	-
Laminectomy lumbar fusion/Instrumentation open	43 (15.5)	181 (16.3)	-
Laminectomy lumbar open	33 (11.9)	128 (11.6)	-
TLIF open	26 (9.4)	92 (8.3)	-
PCF open	16 (5.8)	70 (6.3)	-

⁎ Standardized mean difference reference group is GLP-1 RA cohort. ACDF, anterior cervical discectomy and fusion; BMI, body mass index; GLP-1 RA, glucagon-like peptide-1 receptor agonist; PCF, posterior cervical fusion SGLT-2, sodium-glucose cotransporter-2; TLIF, transforaminal lumbar interbody fusion.

In the GLP-1 cohort, diabetes was present in 198 patients (71.5%), hypertension in 162 patients (58.5%), hyperlipidemia in 98 patients (35.4%), cardiovascular disease in 53 patients (19.1%), kidney disease in 46 patients (16.6%), and anxiety in 34 patients (12.3%). Among the matched controls, diabetes was diagnosed in 808 patients (72.9%), hypertension in 666 patients (60.1%), hyperlipidemia in 391 patients (35.3%), cardiovascular disease in 200 patients (18.1%), kidney disease in 170 patients (15.3%), and anxiety in 133 patients (12%). Among the GLP-1 cohort, 112 patients (40.4%) utilized insulin, 77 (27.8%) used SGLT-2 inhibitors, 47 (17.0%) used sulfonylureas, and 166 (59.9%) were on metformin therapy. In the control group, 366 patients (33%) utilized insulin, 131 (11.8%) used SGLT-2 inhibitors, 159 (14.4%) used sulfonylureas, and 621 (56%) utilized metformin therapy. The SMD values ranged from 0 (hyperlipidemia) to 16% (use of SGLT-2 inhibitor), with all other variables under 10% SMD.

### GLP-1 receptor agonist matched cohort analysis

Table 3 compares perioperative and postoperative outcomes between the GLP-1 RA and matched control cohorts. Patients prescribed GLP-1 RAs had a significantly shorter postoperative LOS compared to controls (GLP-1 RA median: 3 days [IQR: 2–6] vs. Control median: 4 days [IQR: 2–7]; p = .036). The mean LOS was also lower in the GLP-1 RA group (5.27 days vs. 6.35 days), corresponding to an approximate 17% reduction, as estimated by linear regression (coefficient: −1.08 days; 95% CI: −2.08 to −0.07; p = .036). No significant differences were observed in OPR time (GLP-1 RA median: 273 minutes [IQR: 193–413] vs. Control: 283 minutes [IQR: 209–405]; p = .705), 90-day reoperation rates (OR: 1.36; 95% CI: 0.88–2.06; p = .153), 90-day readmission rates (OR: 1.03; 95% CI: 0.75–1.39; p = .873), or nonroutine discharge rates (OR: 0.94; 95% CI: 0.64–1.36; p = .764).

**Table 3:** Comparison of perioperative and postoperative metrics between overall GLP-1 RA cohort and propensity score matched controls.

Outcome variable	GLP-1 RA users (*n* = 277)	Matched controls (*n* = 1,108)	OR	95% CI	p-value
Continuous metrics	Median (IQR)	Median (IQR)			
LOS	3 (2–6)	4 (2–7)	-	-	.036*
OPR minutes	273 (193–413)	283 (209–405)	-	-	.705
Binary metrics	n (%)	n (%)			
Reoperation rate	32 (11.6)	97 (8.8)	1.36	(0.88, 2.06)	.153
Readmission rate	65 (23.5)	255 (23.0)	1.03	(0.75, 1.39)	.873
Nonroutine discharge	40 (14.4)	168 (15.2)	0.94	(0.64, 1.36)	.764

⁎ Demonstrates significant statistical difference, p < .05. Shapiro-Wilks test run to confirm non-normal distribution. Mann-Whitney U Test run to compare distributions. Logistic regression used for binary outcomes to estimate Odds Ratios (ORs) and 95% Confidence Intervals. GLP-1, glucagon-like peptide-1 receptor agonist; IQR, inter-quartile range; OR, odds ratio; OPR, operating room; CI, confidence interval; LOS, length of stay.

Tables 4 and 5 summarize perioperative outcomes between GLP-1 RA users and matched controls across 4 major procedural subcohorts. Patients undergoing lumbar fusion while prescribed GLP-1 RAs had a significantly shorter postoperative LOS compared to controls (GLP-1 RA median: 4.0 days [IQR: 3.0–5.25] vs. Control median: 4.0 days [IQR: 3.0–7.0]; p = .007). Additionally, patients undergoing lumbar laminectomy experienced shorter OPR times in the GLP-1 RA group (GLP-1 RA median: 161.5 minutes [IQR: 109–247] vs. Control median: 217.5 minutes [IQR: 157–250]; p = .021). Among patients undergoing ACDF or ACCF, GLP-1 RA use was associated with higher odds of 90-day reoperation (OR: 3.32; 95% CI: 1.15–10.3; p = .029). No other significant differences were observed in LOS, OPR time, or binary postoperative outcomes across the remaining subcohorts.

**Table 4:** Comparison of continuous perioperative metrics between GLP-1 RA users and propensity-matched controls across major procedural subcohorts.

Subcohort	Outcome variable	GLP-1 RA median (IQR)	No GLP-1 RA median (IQR)	p-value
ACDF + ACCF	LOS	2.0 (1–4.75)	2.0 (1–3)	.958
	OPR minutes	229.5 (177–332)	236 (189–307)	.817
Lumbar laminectomy	LOS	2.0 (1–4.75)	3.0 (1–6)	.696
	OPR minutes	161.5 (109–247)	217.5 (157–250)	.021*
Lumbar fusion	LOS	4.0 (3–5.25)	4.0 (3–7)	.007*
	OPR minutes	339.5 (227–446)	334 (258–463)	.411
Posterior cervical fusion	LOS	6.0 (4–8)	7.0 (4–11)	.209
	OPR minutes	390.0 (305–450)	394 (289–450)	.945

⁎ Demonstrates significant statistical difference, p < .05. ACDF, anterior cervical discectomy and fusion; ACCF, anterior cervical corpectomy and fusion; GLP-1 RA, glucagon-like peptide-1 receptor agonist; IQR, interquartile range; LOS, length of stay; OPR = operating room. Mann-Whitney U Test conducted.

**Table 5:** Comparison of binary perioperative metrics between GLP-1 RA users and propensity-matched controls across major procedural subcohorts.

Subcohort	Outcome variable	Odds ratio (95% CI)	p-value
ACDF + ACCF	Nonroutine discharge	0.70 (0.22, 1.91)	.507
	Readmission	1.27 (0.55, 2.84)	.559
	Reoperation	3.32 (1.15, 10.3)	.029*
Lumbar laminectomy	Nonroutine discharge	0.83 (0.25, 2.41)	.747
	Readmission	0.83 (0.32, 2.02)	.691
	Reoperation	0.65 (0.17, 2.00)	.480
Lumbar fusion	Nonroutine discharge	0.59 (0.28, 1.15)	.136
	Readmission	0.70 (0.40, 1.19)	.192
	Reoperation	0.99 (0.48, 1.96)	.980
Posterior cervical fusion	Nonroutine discharge	2.15 (0.79, 5.83)	.131
	Readmission	1.67 (0.67, 4.14)	.270
	Reoperation	1.06 (0.21, 4.35)	.940

⁎ Demonstrates significiant statistical difference, p < .05. ACDF, anterior cervical discectomy and fusion; ACCF, anterior cervical corpectomy and fusion; CI, confidence intervals; GLP-1 RA, glucagon-like peptide-1 receptor agonist. Logistic regression used to estimate Odds Ratios (ORs) and 95% Confidence Intervals.

## Discussion

In this retrospective study, preoperative GLP-1 RA use was associated with a significantly shorter postoperative LOS compared to matched controls, particularly among patients undergoing lumbar fusion. No significant differences were observed in OPR time, 90-day reoperation rates, 90-day readmission rates, or nonroutine discharge rates among the main cohorts. As the prevalence of obesity and DM continue to rise [27,28], along with increasing rates of elective spine surgery [29,30] and GLP-1 RA prescriptions [31], spinal surgeons are likely to encounter these medications more frequently. Understanding their potential influence on perioperative outcomes is essential. Our findings can help guide preoperative counseling and optimization strategies for patients with obesity and diabetes undergoing spinal decompression and/or fusion.

Several factors may have contributed to the decreased LOS in the GLP-1 RA group, including the drug’s ability to decrease neuroinflammation and pain perception. GLP-1 RAs have demonstrated neuroprotective effects following spinal cord injury by decreasing cell apoptosis and reactive oxygen species (ROS) through inhibition of the mTOR pathway and neuron autophagy [32]. These mechanisms may be linked to the to the drug’s anti-inflammatory effects, such as decreasing levels of IL-6, TNF-a, and increasing levels of IL-10 [33]. GLP-1 RAs have also shown potential in reducing pain hypersensitivity in animal models of osteoarthritis and diabetes-induced neuropathic pain [34]. Together, these mechanisms may potentially contribute to decreased immediate postoperative pain and shortened length of stay observed in the GLP-1 RA group, though this remains a hypothesis warranting further investigation.

Existing literature demonstrates mixed findings regarding the impact of GLP-1 RAs on spine surgery outcomes. Seddio et al. [23] reported that GLP-1 RA use following single-level posterior lumbar fusion was associated with a reduction in 90-day adverse events, although readmission rates were unaffected. Our findings are consistent with this literature, as we observed a statistically significant reduction in LOS among patients undergoing lumbar fusion who were prescribed GLP-1 RAs preoperatively, supporting the potential perioperative benefit of these medications in lumbar fusion procedures. Conversely, Tao et al. [24] found no significant differences in short-term outcomes between patients who took GLP-1 RAs and those who did not prior to cervical decompression and fusion surgery. Similarly, our subcohort analysis revealed no difference in LOS, OPR time, 90-day readmission rate, and nonroutine discharge rate for ACDF and ACCF patients. Interestingly, we did observe an unexpected increase in 90-day reoperation rates for GLP-1 RA users within this procedural subgroup, suggesting that the effect of GLP-1 RAs may vary by spinal region and warrants further investigation.

Beyond procedure-specific findings, our study identified a statistically significant reduction in LOS across the entire cohort, with GLP-1 RA use associated with an approximate 17% decrease in postoperative LOS. Although the absolute reduction was modest, even small improvements in LOS can have meaningful financial implications, particularly in the setting of spinal fusion procedures, which represented a substantial portion of our cohort and rank among the top 3 most expensive operations reimbursed by Medicare [[35], [36], [37]]. Given the anticipated rise in volume and cost of spinal fusions over the next 2 decades [30,36,38], even incremental reductions in LOS may translate to substantial cost savings for healthcare systems. This finding is particularly relevant as healthcare increasingly shifts toward value-based care and bundled payment models, where reducing resource utilization without compromising patient outcomes is a key priority.

Roughly 8% of individuals with diabetes or obesity in our database were prescribed a GLP-1 RA, which is lower than the national average for individuals with these conditions. Studies have shown that GLP-1 RA prescription rates are significantly lower among minority groups, including Asians, African Americans, and Hispanics, compared to White individuals [39]. Additionally, patients with higher household incomes (>$100,000) tend to have higher GLP-1 RA utilization rates [39]. Notably, the majority of our study population consisted of minority ethnic groups, who exhibited high rates of comorbid conditions such as hypertension (>60%) and diabetes (>70%). Our findings highlight the disparity in GLP-1 RA access and underscore the importance of making this effective medication more affordable and widely available to underserved populations with high rates of chronic diseases.

This study has several important limitations. First, its retrospective design limits our ability to establish causation, as we can only report an association between preoperative GLP-1 RA use and surgical outcomes. As with all retrospective studies, there is a risk of residual confounding from unmeasured variables that may influence both medication use and postoperative outcomes. Second, while we controlled for major diabetes and obesity medications, we lacked granular information on the timing, dosage, and duration of GLP-1 RA use, and our cohort included several different GLP-1 RA formulations with varying pharmacologic properties. This is noteworthy as GLP-1 RAs vary significantly in their half-lives, side-effect profiles, methods of administration, and efficacy, which could influence their effect on perioperative and short-term surgical outcomes [40]. Third, our analysis was limited to 90-day outcomes and did not include specific postoperative complication data (e.g., infections, sepsis, DVT, hematomas, or neurological deficits), which could have further contextualized our findings.

Furthermore, GLP-1 RAs have also been shown to improve bone density and bone quality in obese patients undergoing weight loss [41], which may be relevant to spinal fusion healing. However, we lacked data on radiographic fusion status or patient-reported outcome measures (e.g., VAS, ODI, NDI), limiting our ability to assess long-term outcomes. Finally, to maximize statistical power, we combined various spinal decompression and fusion procedures in the primary analysis, introducing some procedural heterogeneity. To address this, we performed a supplementary subcohort analysis stratified by procedure type to better characterize procedure-specific effects which we believe helps characterize the findings more clearly in our larger cohort. Future prospective studies with longer follow-up, detailed medication data, and additional clinical endpoints, including fusion rates and patient-reported outcomes, will be essential to fully understand the impact of GLP-1 RA use in spinal decompression and fusion.

## Conclusion

In this propensity-matched cohort study, preoperative GLP-1 RA use was associated with a significant reduction in postoperative LOS, particularly among patients undergoing lumbar fusion. No significant differences were observed in OPR time, 90-day reoperation rate, 90-day readmission rate, or nonroutine discharge rate. While the absolute reduction in LOS was modest, even small improvements may have important implications given the high costs associated with spinal fusion and the ongoing shift toward value-based care. These findings may help guide spine surgeons when counseling patients using GLP-1 RAs preoperatively. Future prospective studies should aim to incorporate long-term outcomes such as radiographic fusion status, patient-reported outcome measures, and complication rates, and importantly, further delineate procedure-specific effects of GLP-1 RA use on surgical outcomes.

## Data availability

The source data and source code underlying all models, tables, or figures, along with any additional information, may be requested from the corresponding author. The data used for the current project were collected from our institutional clinical databases. We comply with all relevant ethical regulations. Access to the datasets generated during the current study can be provided upon reasonable request, subject to approval by the institutional review board (IRB). This study was approved by the Albert Einstein College of Medicine IRB. As this study did not involve direct patient intervention or interaction, informed consent was not required.

## Funding

This study did not receive any specific funding or sponsorship in the public, commercial, or nonprofit sectors for the design and conduct of the study; collection, analysis, or interpretation of data; or preparation or review of this manuscript.

## Declaration of competing interests

The authors declare that they have no known competing financial interests or personal relationships that could have appeared to influence the work reported in this paper.
